# Early bilateral hippocampal lesions in transient global amnesia: Evidence against delayed ischemia?

**DOI:** 10.5348/ijcri-201467-CL-10055

**Published:** 2014

**Authors:** Kelsey Flynn, Pascale Lavoie, Robert Laforce

**Affiliations:** 1 BSc, University of British Columbia, Vancouver, Canada; 2 MD, Faculté de Médecine, Université Laval, Québec, Canada; 3 MD, PhD, Faculté de Médecine, Université Laval, Québec, Canada; Clinique Interdisciplinaire de Mémoire, Département des Sciences Neurologiques, Hôpital de l’Enfant-Jésus, Québec, Canada

## CASE REPORT

A 65-year-old right-handed female was admitted to neurology after she suddenly began experiencing problems with her memory. Her past medical history was significant for osteoarthritis and a fractured clavicle, but there were no vascular risk factors, head trauma, migraine or epilepsy. She did not smoke, occasionally drank alcohol and denied illicit drug use. Her mother had passed away at 98 years of age without any sign of Alzheimer’s disease but one of her aunts was diagnosed with Alzheimer’s disease. She has four siblings, all were in good health, except for one brother with cardiac problems.

On the day of the event, at 11:15 a.m. she began showing symptoms after a short bout of intense running for almost six minutes. She reported a vague sensation of confusion as to where she was and what she was doing, but nonetheless called her husband to pick her up. When he arrived, he noted her confusion in space and time, and she admitted to feeling dizzy. Upon arrival at their home, her confusion persisted. For example, she had forgotten about her sister’s birthday, and that they were renovating their kitchen. She repeatedly asked her husband “Why is there a present on the counter?”, or “Why is our kitchen such a mess?” After a few hours, she returned to normal level of functioning with no recollection of what had just happened.

**Figure 1:**
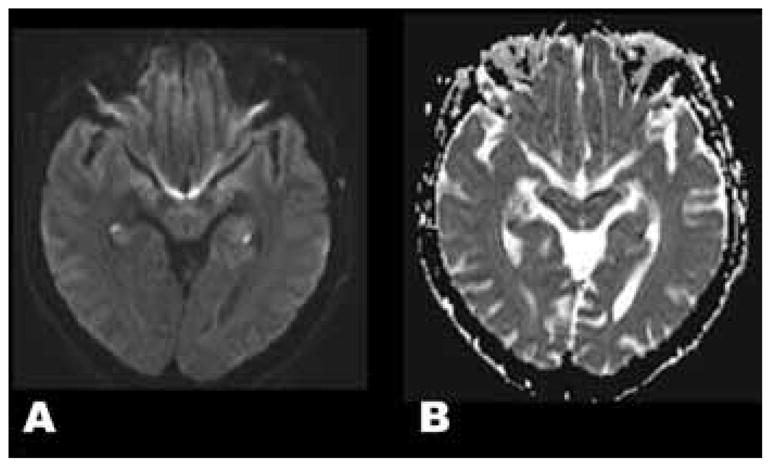
Magnetic resonance imaging scan of the brain (A) Showing bilateral hyperintense posterior portions of the hippocampi, (B) Hypointense apparent diffusion coefficient map in similar brain loci, both compatible with ischemic lesions. Follow-up Magnetic resonance imaging conducted six weeks later showed complete resolution of the lesions.

Neurological examination was normal. Blood pressure was 122/68 mmHg, heart rate 76 bpm. Basic blood work, ions, urea/creatinine, TSH, calcium/magnesium/phosphorus, B12/folates were unremarkable. She scored 26/30 on a brief cognitive screening measure, losing four points in the free recall section, but showing intact recognition with verbal cues. The rest of her cognitive examination was normal. Magnetic resonance imaging (MRI) scan of the brain conducted 19 hours after symptom onset revealed bilateral lesions of the hippocampi (Figure 1).

## DISCUSSION

The patient was diagnosed with transient global amnesia (TGA), a benign syndrome characterized by the sudden onset of severe anterograde and mild retrograde amnesia [1]. Transient global amnesia is generally accompanied by repetitive questioning and resolves within 24 hours, leaving no sequelae. Recurrence is rare. Several possible etiologies have been suggested as underlying mechanisms for TGA including migraine-related mechanisms, venous-flow abnormalities, epileptic seizures, and ischemic events [2–4]. However, the fundamental etiology of TGA remains unclear and may be highly heterogeneous.

Recent neuroimaging studies have revealed that a large portion of individuals with TGA present with small, reversible, restricted lesions on diffusion MRI [5]. This has been used to support an underlying ischemic event that could be the result of venous overflow. Furthermore, these lesions often tend to appear when patients are scanned after a 48-hour delay, hence supporting the delayed ischemic hypothesis [5]. Interestingly, our patient’s ischemic event was detected in the acute phase (0–24 hours) of TGA where most other authors have failed to report any changes. Whether this is attributable to lesion size is unclear, but nonetheless does not support the notion of delayed ischemia.

We herein report a case of transient global amnesia (TGA) secondary to bilateral ischemic hippocampal lesions 19 hours after symptom onset. In accord with the hypothesized etiology of venous overflow, our findings replicate other studies showing reversible ischemia in hippocampal regions associated with this syndrome. Interestingly, lesions to other structures known to be involved in declarative memory (e.g., thalamus, cingulate gyrus, and basal ganglia) have been associated with TGA [6]. In addition to being rare because of their bilateral nature, the lessions shown here are unique in that very few authors have reported such changes in the acute phase of TGA. Moreover, they raise the possibility that delayed ischemia may not be the sole mechanism behind TGA. Indeed, ischemia may be present early in the process but simply not detected [5].

## CONCLUSION

Newly developed imaging techniques such as functional Magnetic resonance imaging scan may allow earlier detection of cerebral changes associated with transient global amnesia. This in turn may help clinicians in the differential diagnosis of other transient amnestic syndromes, as well as more rapid targeted interventions.
